# Covalent Modification of Mutant Rat P2X2 Receptors with a Thiol-Reactive Fluorophore Allows Channel Activation by Zinc or Acidic pH without ATP

**DOI:** 10.1371/journal.pone.0047147

**Published:** 2012-10-24

**Authors:** Shlomo S. Dellal, Richard I. Hume

**Affiliations:** 1 Department of Molecular, Cellular and Developmental Biology, University of Michigan, Ann Arbor, Michigan, United States of America; 2 Department of Neurobiology, University of California Los Angeles, Los Angeles, California, United States of America; The Ohio State University, United States of America

## Abstract

Rat P2X2 receptors open at an undetectably low rate in the absence of ATP. Furthermore, two allosteric modulators, zinc and acidic pH, cannot by themselves open these channels. We describe here the properties of a mutant receptor, K69C, before and after treatment with the thiol-reactive fluorophore Alexa Fluor 546 C_5_-maleimide (AM546). *Xenopus* oocytes expressing unmodified K69C were not activated under basal conditions nor by 1,000 µM ATP. AM546 treatment caused a small increase in the inward holding current which persisted on washout and control experiments demonstrated this current was due to ATP independent opening of the channels. Following AM546 treatment, zinc (100 µM) or acidic external solution (pH 6.5) elicited inward currents when applied without any exogenous ATP. In the double mutant K69C/H319K, zinc elicited much larger inward currents, while acidic pH generated outward currents. Suramin, which is an antagonist of wild type receptors, behaved as an agonist at AM546-treated K69C receptors. Several other cysteine-reactive fluorophores tested on K69C did not cause these changes. These modified receptors show promise as a tool for studying the mechanisms of P2X receptor activation.

## Introduction

P2X receptors are ATP-gated ion channels involved in a wide array of functions. Seven P2X genes (P2X1–P2X7) are present in mammals. The P2X2 protein shows widespread expression in the central nervous system, including the hippocampus and cerebellum [1] as well as in sensory and autonomic ganglia, and has been implicated in nociception [2], [3], taste [4], peristalsis of the gut [5], [6], and ventilatory responses to hypoxia [7]. P2X2 receptors assemble as trimers, either as homotrimers [8], [9], [10], [11] or as heterotrimers with a subset of the other P2X subunits [12], [13], [14].

The open probability of rat P2X2 channels (rP2X2) in the absence of ATP is undetectably low [15], [16]. Furthermore, when the concentration of ATP is low, the ATP responses of rP2X2 channels are substantially increased by acidic pH [17], [18], [19], [20] and by extracellular zinc in the range of 5–100 µM [17], [20]. However, these modulatory agents have little or no ability to open wild type channels on their own [21], [22]. The zinc binding site responsible for potentiation has been determined to lie at the interface between adjacent subunits and includes one histidine on each side of the interface [23].

A decade of mutagenesis studies carried out in several labs [24] uncovered candidates for participation in ATP binding, and suggested that like zinc, ATP binds to the receptor between receptor subunits [25], [26]. Two highly conserved lysines, K69 and K308 (rP2X2 numbering), received particularly strong support as ATP binding residues. Mutation of either of these lysines to alanine in several P2X receptor subtypes resulted in a >100 fold reduction in agonist potency, with no apparent decrease in surface expression [27], [28], [29], [30]. Furthermore, mutation of a neighboring residue, I67, to cysteine yielded a receptor with no overt phenotype but treatment of these receptors with the thiol-reactive drug sodium (2-sulfonatoethyl) Methanethiosulfonate (MTSES) caused a rightward shift in the ATP concentration-response curve with no decrease in peak current amplitude, which suggested that the modified residue was impeding access of ATP to its binding partners [27]. In P2X1, the mutant homologous to K308C exhibited reduced ^32^P 2-azido ATP binding and treatment with 2-Aminoethyl Methanethiosulfonate Hydrobromide (MTSEA) caused a significant leftward shift in the concentration-response curve, presumably by restoring the positive charge [31]. These inferences were strongly supported by the initial crystallization of a zebrafish P2X receptor, zP2X4.1, even though that structure was obtained in the unliganded state [32] and confirmed by the very recent crystallization of zP2X4.1 bound to ATP [33]. Lysine 70 (the zP2X4.1 homolog of K69) plays a key role by interacting with the α, ß and γ phosphates of ATP and with C8 and N6 of the adenine, while K316 (the zP2X4.1 homolog of K308) on the adjacent subunit interacts with the ß and γ phosphates.

In the course of experiments to further characterize the ATP binding site of rP2X2 receptors, we discovered that treatment of K69C mutant receptors with the cysteine-reactive fluorophore Alexa Fluor 546 C_5_-maleimide (AM546) had several interesting effects. AM546 caused a small but significant activation of these channels in the absence of exogenous ATP, and either micromolar zinc or acidic pH elicited inward currents from these chemically modified receptors in the absence of exogenous ATP. Because these modified receptors might potentially be useful for understanding the gating of P2X2 receptors, we characterized their properties in detail.

## Materials and Methods

### Engineering of Mutant P2X2 Constructs

A plasmid encoding rP2X2 was obtained from Dr. D. Julius, University of California, San Francisco. Mutations were generated using the QuikChange Site-Directed Mutagenesis kit (Stratagene, La Jolla, CA). The sequences of all constructs were confirmed by DNA sequencing (University of Michigan DNA Sequencing Core).

### Expression of P2X2 Receptors

This study was carried out in strict accordance with the recommendations in the Guide for the Care and Use of Laboratory Animals of the National Institutes of Health. The protocol was approved by the University Committee on the Use and Care of Animals of the University of Michigan (Protocol 08396-4). The rP2X2 receptors were expressed in defolliculated stage V-VI *Xenopus laevis* oocytes. Oocytes were harvested using procedures that have been described in detail previously [34].

RNAs encoding wild type and mutant rP2X2 receptors were synthesized using the mMessage mMachine T7 kit (Life Technologies, Grand Island, NY). Each oocyte was injected with 50 nl of RNA (10–20 ng/µl). Two-electrode voltage clamp experiments were performed 2–5 days after RNA injection. Except where indicated otherwise, recordings were made at a holding potential of −50 mV. Recording electrodes were pulled from thin walled borosilicate glass and had resistances of 0.2–1 MΩ. Currents were recorded with a Turbo TEC-03 or TEC-10 voltage clamp amplifier (npi electronic GmBH, Tamm, Germany). Data acquisition was performed using a Digidata 1322A interface controlled by pCLAMP 9 or 10 (Molecular Devices, Sunnyvale, CA).

To regulate the delivery of most test solutions into the recording chamber, we used a gravity fed valve array. The solution flowing out of an 8-way mini-manifold (MP-8, Warner Instruments, Hamden CT) connected to the recording chamber was controlled by switching the input lines on and off with a computer controlled array of pinch valves (VM8 with BPS-8 controller, ALA Scientific Instruments, Farmingdale, NY). At the flow rates typically used, the time constant for solution exchange in the recording chamber was approximately 3 seconds. To conserve materials used in studies of the effect of modification of accessible cysteines, recordings were made from oocytes while the flow through the recording chamber was stopped and a concentration of reagent sufficient to cause maximum modification within a 2 minute incubation was added from a P-200 pipetter in a volume sufficient to cause a nearly complete solution change within a few seconds. At the end of the incubation period, a wash of at least 30 s occurred before recording resumed.

### Solutions

The external recording solution contained (in mM): 90 NaCl, 1 KCl, 1.7 MgCl_2_, and 10 HEPES, pH 7.5. In some experiments we varied the pH of the recording solution. This was done by adding either HCl or NaOH. Recording electrodes were filled with 3 M KCl. Disodium ATP (Sigma-Aldrich, St. Louis, MO) was prepared as a 100 mM stock and stored at −20°C. For recording, ATP solutions were made by diluting the stock in external recording solution. The ATP concentrations of all recording solutions were verified by spectroscopic measurement at 259 nm (Biomate 3, Thermo Electron Corporation, Madison, WI). Zinc chloride was prepared as a 100 mM stock in double-distilled H_2_O that was acidified with 0.01 M HCl to prevent precipitation. Except where noted otherwise, the pH of ATP solutions with and without zinc was adjusted to 7.5 prior to recording. All ATP recording solutions were used within 48 h.

All thiol-reactive reagents were prepared as stock solutions in dimethyl sulfoxide (DMSO) and stored frozen in aliquots at −20°C. The final working strength solutions were diluted with external recording solution on the day of the experiment and kept on ice until just before they were added to the oocyte incubation chamber. Fluorescent reagents were also protected from light. For each reagent and each mutant, pilot experiments were carried out to find the combination of concentration and exposure time that produced a maximal response, and this duration of exposure was used in subsequent experiments. The sulfhydryl-reactive reagents MTSEA (2-Aminoethyl Methanethiosulfonate Hydrochloride), MTSES (Sodium (2-Sulfonatoethyl)Methanethiosulfonate), MMTS (Methyl Methanethiosulfonate) and MTSET ([2-(Trimethylammonium)ethyl] Methanethiosulfonate Chloride), all from Toronto Research Chemicals (North York, ON, Canada) were prepared as 100 mM stocks. The sulfhydryl-reactive fluorophores Alexa Fluor 488 C_5_-maleimide, Alexa Fluor 546 C_5_-maleimide, Alexa Fluor 568 C_5_-maleimide and Alexa Fluor 594 C_5_-maleimide (Life Technologies, Grand Island, NY) were prepared as 10 mM stocks. Suramin (Sigma Life Sciences, St. Louis MO) was prepared at 200 µM in external recording solution and all other suramin solutions were prepared by diluting this stock solution.

### Data Analysis

Data analysis was done using Clampfit, Excel and SigmaPlot 9.0. To test for statistical significance we used the two tailed *t*-test function of Excel, with significance taken to be p<0.05. When responses of individual oocytes studied before and after treatments were analyzed, the paired test was used, otherwise the unpaired test was used.

Concentration-response plots were fit to the three-parameter Hill equation using the nonlinear curve fitting program of SigmaPlot 9.0. For displaying average data, the points from each cell were normalized to between 0 and 100% based on the maximum value of the fitted curve for that cell. The scaled data were then averaged across multiple cells, and plotted with error bars indicating the standard error of the mean. The lines fit to the data indicate the average parameters of the individual fits.

Electrophysiological traces show inward currents as downward deflections and when the magnitudes of inward currents are reported in the text, they are preceded by minus signs to distinguish them from outward currents. However, when data are presented as X-Y or bar graphs, the amplitudes of inward currents are usually plotted as upward deflections to allow easy visual assessment of the data. The one exception to this convention is noted in the appropriate figure legend.

## Results

### MTSEA Partially Restored ATP Responses in K69C and K308C Mutants

**Figure 1:**
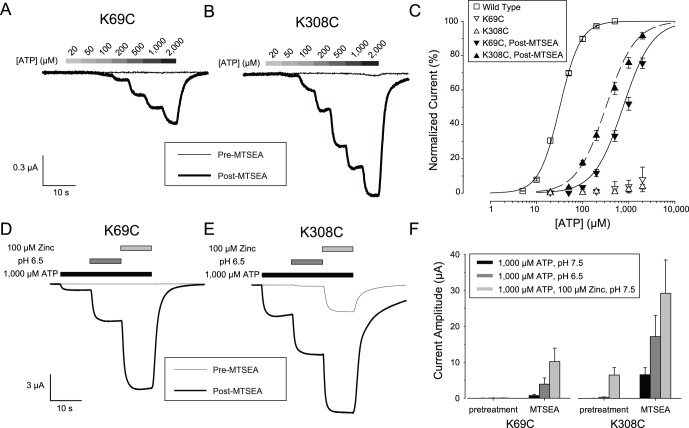
Activation of K69C and K308C mutant receptors by MTSEA. A: Response of an oocyte expressing K69C to ATP before (thin line) and after (thick line) treatment with MTSEA. B: Similar data for an oocyte expressing K308C. The scale bar is the same as in A. C: ATP concentration-response relations for a series of oocytes studied as in A and B. The error bars represent standard error of the mean and are smaller than the symbols for some points. (N = 5–6). D–E: Response to zinc or acid jumps in the presence of 1,000 µM ATP of oocytes expressing K69C or K308C before (thin line) and after (thick line) treatment with MTSEA. F: Quantitative characterization of a series of experiments studied as in D and E (N = 3). In this figure, and most others, the average inward current is plotted as an upward bar. The exception to this convention is noted in the legend for the relevant figure.

To probe the mechanism of receptor activation, we introduced a cysteine at either position 69 or 308 of rP2X2, expressed the mutant receptors in *Xenopus* oocytes and tested reagents expected to be able to react covalently with free cysteines. We suspected that the unmodified K69C mutant might be unresponsive to ATP because when expressed in HEK293 cells the K69A mutant did not respond to 100 µM ATP [27]. More recent work however, has indicated that the K69A mutant has some residual affinity for ATP at very high concentrations [25]. When oocytes expressing the K69C mutant were voltage clamped at −50 mV and exposed to 100 µM ATP, no current was evoked. When 1,000 µM ATP was applied, many oocytes still showed no current but some oocytes responded with very small inward currents (mean inward current −8±1 nA, N = 5). Oocytes injected with the same concentration of RNA encoding wild type rP2X2 gave inward currents of over −20 µA in response to 1,000 µM ATP. The failure of oocytes expressing K69C to respond to ATP was not due to the absence of receptors on the plasma membrane because addition of MTSEA, a small, positively-charged, sulfhydryl-reactive reagent, allowed these mutant receptors to respond to ATP with large inward currents (Fig. 1A). The implication of this finding is that MTSEA restored a positive charge at the mutated residue and hence partially restored receptor function. Similar results were observed when the cysteine mutant of the other lysine implicated in ATP binding (K308C) was tested in the same manner (Fig. 1B). Recovery of receptor function following MTSEA treatment had previously been reported in P2X1 for the mutant analogous to K308C [31]. The MTSEA-modified K69C and K308C receptors both had ATP concentration-response relations right-shifted more than 10 fold as compared to wild type rP2X2 (Fig. 1C), with EC_50_s of 924±102 and 374±35 µM, respectively as compared to the wild type rP2X2 EC_50_ of 32±1 µM. Both wild type rP2X2 and the MTSEA-modified K69C and K308C receptors had Hill coefficients greater than 1 (wild type, 1.9±0.01; K69C, 1.4±0.1; K308C, 1.3±0.1).

We next asked whether the allosteric modulators zinc and acidic pH could potentiate the ATP response of MTSEA-treated oocytes expressing K69C and K308C. When untreated wild type receptors are provided with an EC_10_ concentration of ATP at pH 7.5, addition of 20 µM zinc (a zinc jump) or exposure to pH 6.5 (an acid jump) results in an approximately 8 fold enhancement of the current [17], [20]. For untreated K69C receptors, 1,000 µM ATP elicited no significant responses, even when the solution was supplemented with 100 µM zinc or made acidic, but after MTSEA treatment, zinc and acidic pH both greatly potentiated the K69C response to 1,000 µM ATP (Fig. 1D, F). For untreated K308C receptors, 1,000 µM ATP alone at pH 7.5 produced negligible responses, but in contrast to untreated K69C receptors, both 1,000 µM ATP +100 µM Zinc and 1,000 µM ATP at pH 6.5 elicited easily detectable responses (Fig. 1E, F). After MTSEA treatment, the potentiation of the ATP responses of K308C receptors by zinc and pH were both greatly enhanced (Fig. 1E, F). Uninjected oocytes exposed to a zinc jump or an acid jump had average responses of less than 10 nA (+8.6±1.9 nA for 50 µM zinc; −7.3±5.7 for pH 6.5; N = 14 for each) which were similar in size to the responses to the recording solution alone applied from two different barrels of the solution switcher (+3.1±0.9; N = 48).

To assess the specificity of the effect of MTSEA on the channel properties of K69C and K308C we tested three other cysteine reactive compounds (Fig. S1). We first exposed the oocytes to the test compound (MMTS, MTSES, or MTSET) then washed and applied 1,000 µM ATP. None of these compounds restored the ability of either K69C or K308C to respond to ATP. To verify that the test compounds had successfully modified the receptor, the application of test compound was followed by application of MTSEA (MTSEA challenge), a wash and retesting with ATP. For K69C expressing oocytes, all incubations were 1 minute. Based on the failure of the MTSEA challenge to increase the response to ATP, this was indeed sufficient time for all three test compounds to fully modify the accessible cysteines. The modification rate of K308C expressing oocytes was much slower, and so to produce nearly maximal effects the incubation in the test compound was increased to 15 minutes. MTSES is larger in size than MTSEA and differs in charge (negative rather than positive), MMTS is smaller than MTSEA and uncharged, and MTSET is larger than MTSEA but positively charged, so these results suggest that both the small size and the positive charge of MTSEA are required for the rescue of ATP responses in K69C and K308C.

### Covalent Modification of K69C with AM546 Caused Constitutive Activation of the Receptor

If MTSEA treatment of the K69C and K308C receptors restores a required positive charge to the ATP binding site, then treatment of these positions with larger molecules might make it impossible for ATP to bind. Receptors thus modified would have no ATP responses, unlike unmodified receptors which have measurable, but very small responses to very high ATP. However, when Alexa Fluor 546 C_5_-maleimide (AM546), which has a molecular weight about 4 times greater than MTSEA, was tested on K69C expressing oocytes, the results we obtained were unexpected, and very different from the results obtained from oocytes expressing wild type receptors.

**Figure 2:**
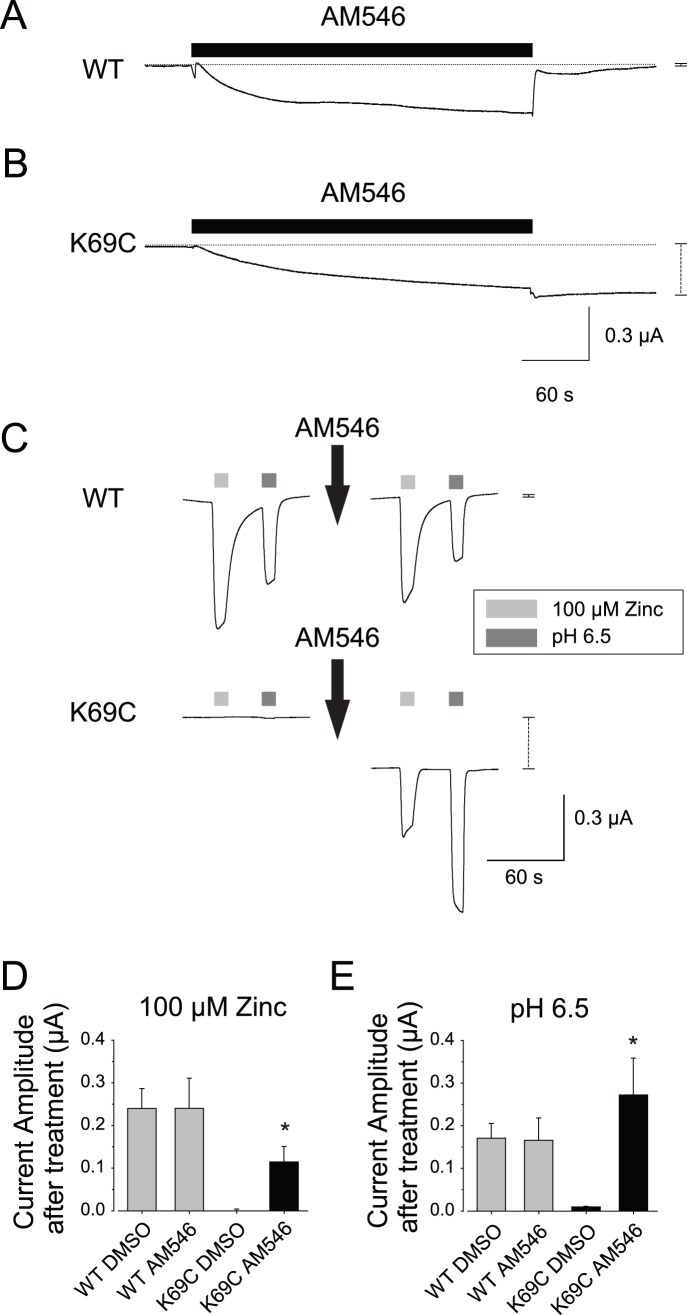
AM546 activated the K69C receptor. A–B: Sample traces from oocytes expressing either wild type rP2X2 (WT) or K69C as they were exposed to 50 µM AM546 for 5 minutes and then washed with recording solution. The horizontal dotted lines indicate the pretreatment holding current levels. The vertical dashed lines to the right of each trace indicate the difference in holding current 2 min after washout from the holding current before exposure to the drug. For wild type the change was so small that the whiskers obscure the amplitude of the dashed line. C: AM546 treatment caused K69C expressing oocytes to become responsive to zinc and acidic pH in the absence of exogenous ATP, but had no effect on responses of wild type receptors. Note the change in holding current after AM546 treatment in K69C expressing oocytes but not wild type oocytes (vertical dashed lines to the right of the traces). D–E: Quantitative characterization of a series of oocytes studied as in C, as well as control oocytes exposed to the vehicle alone (0.5% DMSO) (N = 4–8). Asterisks indicate a statistically significant difference from the DMSO control tested on the same construct.

During exposure of oocytes expressing wild type rP2X2 to AM546 (50 µM, 5 min), we often observed small increases in the magnitude of the holding current, but the inward current was not sustained after the AM546 solution was removed (Fig. 2A). The small responses of oocytes expressing wild type rP2X2 during AM546 treatment may have represented a vehicle effect, as when oocytes expressing wild type rP2X2 were treated with 0.5% DMSO in external recording solution (the same concentration of DMSO as mixed with the AM546 that was applied in the experimental conditions) currents of similar amplitude were observed (average change during AM546 incubation = −43±48 nA (N = 9), average change during DMSO incubation = −210±121 nA, N = 7; average change after AM546 washout = +71±24 nA and after DMSO washout = +36±13 nA). In contrast, when oocytes expressing K69C were treated with AM546, a more negative holding current was sustained after the AM546 was washed out (Fig. 2B). There was considerable variation in the amplitude of the sustained inward current, and in a few cases it exceeded −500 nA. For a set of oocytes from a single frog, sustained inward current after washout of AM546 for K69C expressing oocytes averaged −129±13 nA (N = 9), which was a highly significant increase (p<.001, Paired T-test, final vs. initial holding current).

One potential explanation for the sustained inward current was that covalent binding of AM546 binding to K69C put the receptors into a state from which they sometimes opened in the absence of ATP. An alternative explanation was that AM546 greatly enhanced the sensitivity of the modified K69C receptors to ATP, so that the small amount of ATP that leaks out of oocytes was sufficient to elicit a sustained current. However, enhanced ATP responsiveness cannot be the cause of the sustained current. The basal ATP concentration due to leakage is 0.1–2 µM (see Discussion), but at pH 7.5 the AM546-modified K69C receptors were insensitive to exogenous 100 µM ATP and just barely responsive to 1,000 µM ATP (see section below “ATP responses of K69C expressing oocytes after AM546 treatment”).

To further explore the effects of AM546, we tested two other treatments known to make it easier to open the wild type channels, exposure to micromolar zinc and to acidic pH. Previous work indicates that both modulators require ATP for activity. However, we found that in the absence of exogenous ATP, oocytes expressing wild type rP2X2 at high levels (maximal responses greater than −20 µA in the presence of saturating ATP) usually responded to a zinc jump or an acid jump with inward currents of a few hundred nA (Fig. 2C). The likely explanation is that most oocytes leak enough ATP to cause low level activation of wild type rP2X2. One line of evidence in support of this idea is that the magnitudes of the zinc and pH responses from these oocytes showed a strong correlation with the amplitude of the inward holding current at the start of the recording protocol (I_holding_ vs. I_zinc_, slope = .31 nA/nA, y-intercept = −48 nA, R^2^ = .82; I_holding_ vs. I_pH 6.5_, slope = .46 nA/nA, y-intercept = −57 nA, R^2^ = .76; N = 10). The responses of wild type rP2X2 receptors to zinc and acid jumps were not changed by AM546 treatment (Fig. 2 C–E) as expected because wild type receptors have no free extracellular cysteines. The results for oocytes expressing K69C receptors were very different. No inward current was observed in response to zinc or acid jumps before AM546 treatment or after DMSO controls, but currents of several hundred nA were seen after AM546 treatment (Fig. 2 C–E).

**Figure 3:**
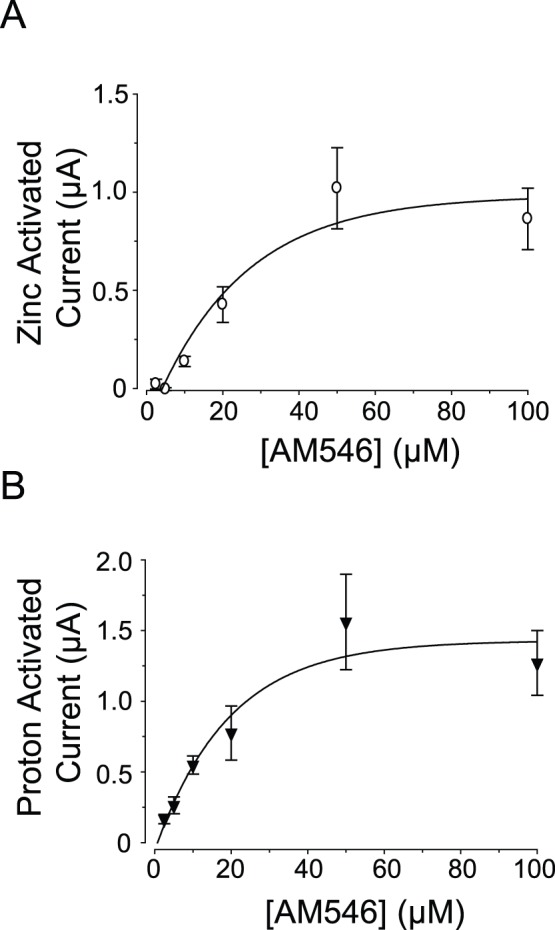
Concentration-response relation of activation of K69C receptors by AM546. A–B: Concentration-response plots of the current activated by 100 µM zinc, and current activated by pH 6.5 after incubation in AM546. The data in the plots were fit with a single-exponential rise to a maximum (N = 5–10).

When the duration of AM546 treatment was held constant at 5 minutes, but the concentration of AM546 was varied so as to produce different numbers of modified receptors, the responses to AM546 of oocytes expressing K69C showed similar concentration dependence regardless of whether the variable being measured was the current in response to a zinc jump or the current in response to an acid jump (Fig. 3). These results also indicate that a 5 minute exposure to 50 µM AM546 was sufficient to have a maximal effect.

### Characteristics of the Channels Activated in K69C Receptors after AM546 Treatment

**Figure 4:**
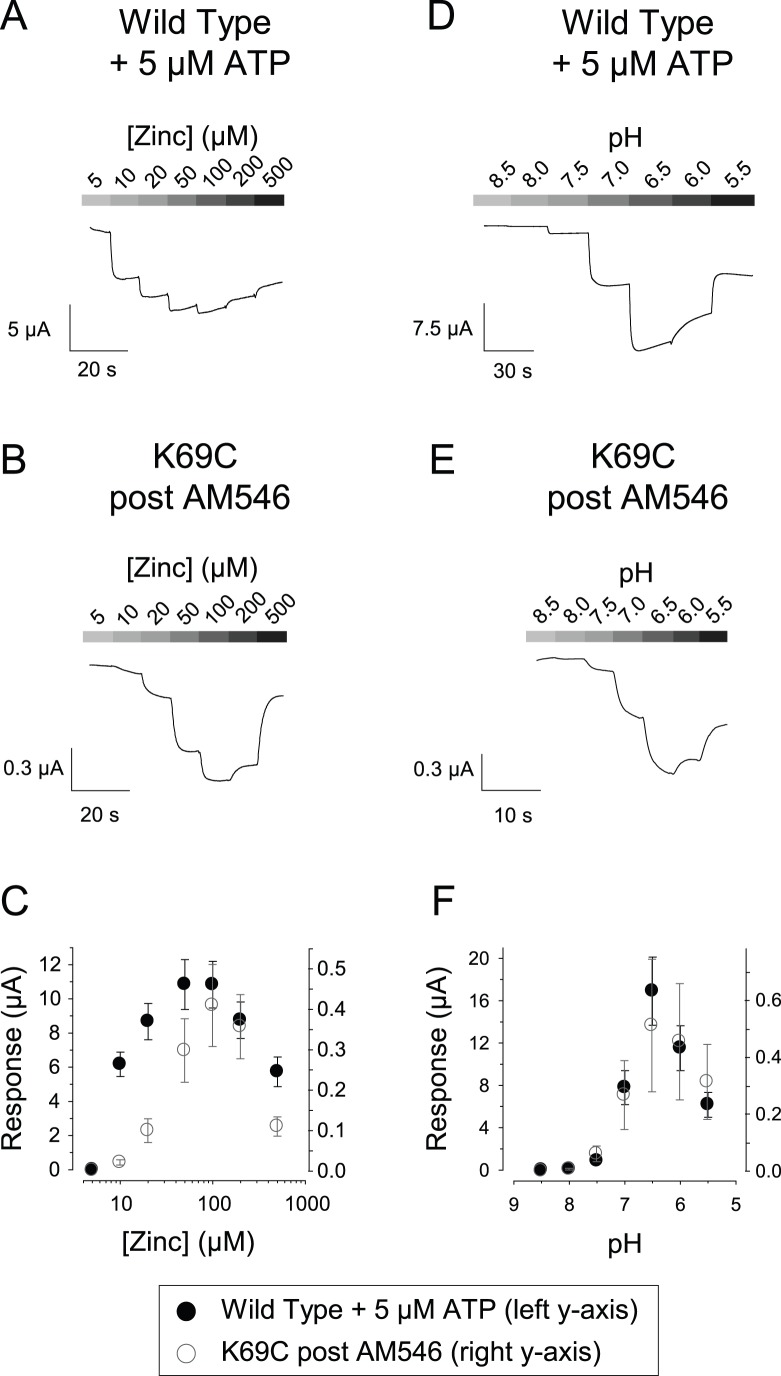
Concentration-response relations for the effects of zinc and pH. A: Effect of increasing zinc concentration on the currents of an oocyte expressing wild type rP2X2 in the continuous presence of 5 µM ATP. There was already a substantial inward current at 5 µM ATP with 5 µM zinc, but for quantifying the magnitude of the effect for a series of similar oocytes, the current with 5 µM zinc was defined as 0, and changes from this level are shown in the trace and the accompanying graph in C. B: Effect of increasing zinc concentration, in the absence of exogenous ATP, on the currents of an oocyte expressing K69C after treatment with AM546. The current with 5 µM zinc was defined as 0. C: Quantification of a series of oocytes studied as in A and B. The data points for wild type rP2X2 (filled circles) are scaled by the left y-axis and the data points for K69C (open circles) are scaled by the right y-axis (N = 5). D: Effect of increasing proton concentration on the currents of an oocyte expressing wild type rP2X2 in the continuous presence of 5 µM ATP. The currents at pH 8.5 were defined as 0. E: Effect of increasing proton concentration, in the absence of exogenous ATP, on the currents of an oocyte expressing K69C after treatment with AM546. The currents at pH 8.5 were defined as 0. F: Quantification of a series of oocytes studied as in D and E. The data points for wild type rP2X2 (filled circles) are scaled by the left y-axis and the data points for K69C (open circles) are scaled by the right y-axis (wild type, N = 7; K69C, N = 3).

For oocytes expressing wild type rP2X2, the effect of zinc jumps and acid jumps is highly dependent on the concentration of zinc or protons, with bell-shaped concentration-response curves produced by a higher affinity potentiating site and a lower affinity inhibitory site [17]. K69C expressing oocytes treated with AM546 also showed bell-shaped concentration-response relations for zinc and pH (Fig. 4), demonstrating that the binding sites for both modulators were left intact following treatment.

A characteristic of the zinc and pH potentiation of wild type rP2X2 receptors is that the binding sites responsible for these phenomena interact allosterically with the ATP binding site [20]. Because the open probability cannot exceed 1, the amount of potentiation possible decreases as the ATP concentration increases. For oocytes expressing wild type rP2X2 tested with a concentration of ATP sufficient to cause a response 10% of the maximum at pH 7.5, addition of zinc (20 µM) or protons (pH 6.5) raised the current to 80–90% of the maximum, so the combination of the two treatments did little more than either alone (Fig. S2A, S2D) [17], [20]. However, by selecting a combination of ATP, zinc, and pH that would be expected to keep the open channel probability relatively low (2 µM ATP, 2 µM zinc, pH 7.2), the combination of both zinc and pH produced a combined response that exceeded the sum of the responses of either treatment alone (Fig. S2B, S2D). For AM546 treated K69C oocytes, the simultaneous application of 100 µM zinc and an acid jump to 6.5, in the absence of exogenous ATP, produced a response that also exceeded the sum of the responses to either alone (Fig. S2C–D). This implies that the maximal open probability reached following AM546 alone is far less than 10%. A second way to reach the same conclusion is to note that the standing inward current of K69C expressing oocytes in response to AM546 alone averaged about −100 nA, while other oocytes from the same batch produced currents of greater than −10 µA when modified by MTSEA and exposed to ATP and zinc (Fig. 1 D, F). This suggests that the activation of K69C by AM546 alone is on the order of 1%.

**Figure 5:**
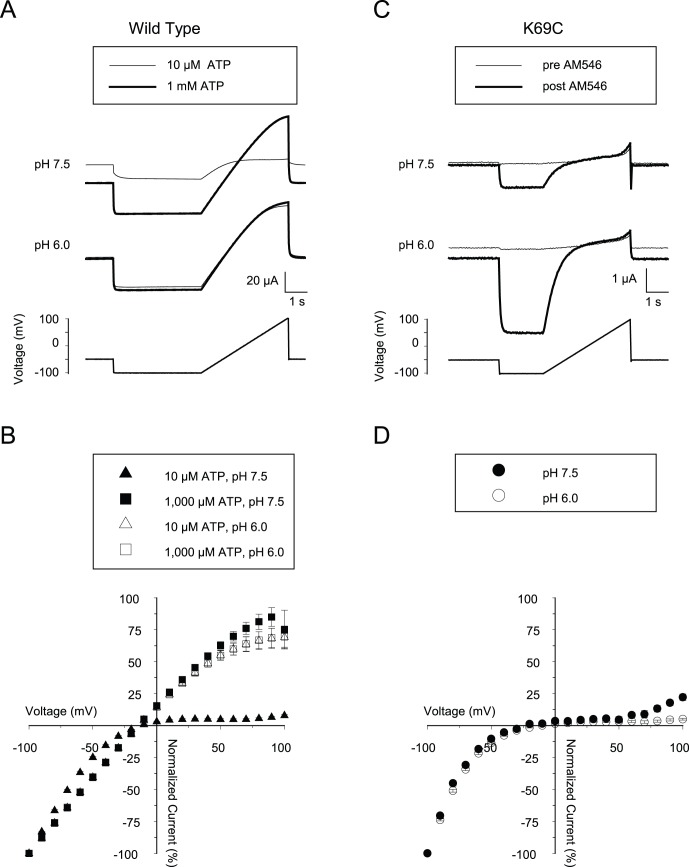
Effects of voltage on wild type rP2X2 and on AM546-treated K69C receptors. A: Representative traces of a rP2X2 expressing oocyte to a 2 s ramp from −100 to +100 mV in the presence of either 10 or 1,000 µM ATP (thin and thick traces, respectively) at pH 7.5 or pH 6.0. ATP was applied beginning approximately 5 seconds before the portion of the traces that are illustrated B: Current-voltage plots of rP2X2 receptor responses based on multiple experiments as in A. Data from ramps in the absence of ATP were subtracted from data in the presence of ATP. (N = 7). C: Representative traces of a K69C expressing oocyte to a voltage ramp at either pH 7.5 or pH 6.0 recording solution, before (thin traces) and after (thick traces) AM546 treatment. D: Current-voltage plots based on multiple experiments as in C. Data from ramps before AM546 treatment were subtracted from data taken after AM546 treatment. (N = 6).

There is also an allosteric interaction between membrane potential and ATP concentration in wild type rP2X2 channels. More negative potentials favor channel opening [16], [34], and so at relatively low concentrations of ATP the currents through wild type rP2X2 channels show strong inward rectification as the cell is depolarized, but the rectification is much less profound at very high ATP concentrations [35], [36]. To compare the extent of inward rectification in AM546-activated K69C channels with ATP-activated wild type rP2X2, we examined the responses to voltage ramps from -100 mV to +100 mV at pH 7.5 and 6.0. For the wild type channels (Fig. 5A) we subtracted the responses to voltage ramps in the absence of ATP from those in the presence of either high (1,000 µM) or low (10 µM) ATP and then normalized all currents to the current at −100 mV to get the I–V relation (Fig. 5B) while for AM546-activated K69C channels (Fig. 5C) we obtained the I–V relation by subtracting the responses prior to treatment from those after AM546, before normalizing (Fig. 5D). For wild type rP2X2 channels, the I–V relations to 10 µM and 1,000 µM ATP applied at pH 6.0 and for 1,000 µM ATP applied at pH 7.5 were nearly identical, while much greater inward rectification was observed for 10 µM ATP at pH 7.5. The inward currents at −100 mV were also much smaller under the latter condition. For K69C expressing oocytes activated by AM546, the inward currents at −100 mV were much larger at the more acidic pH, but the I–V relation was highly inwardly rectifying even at pH 6.0 (Fig. 5D). This result is also consistent with the conclusion that AM546 activated the K69C channels to a limited extent, even at pH 6.0.

### ATP Responses of K69C Expressing Oocytes after AM546 Treatment

**Figure 6:**
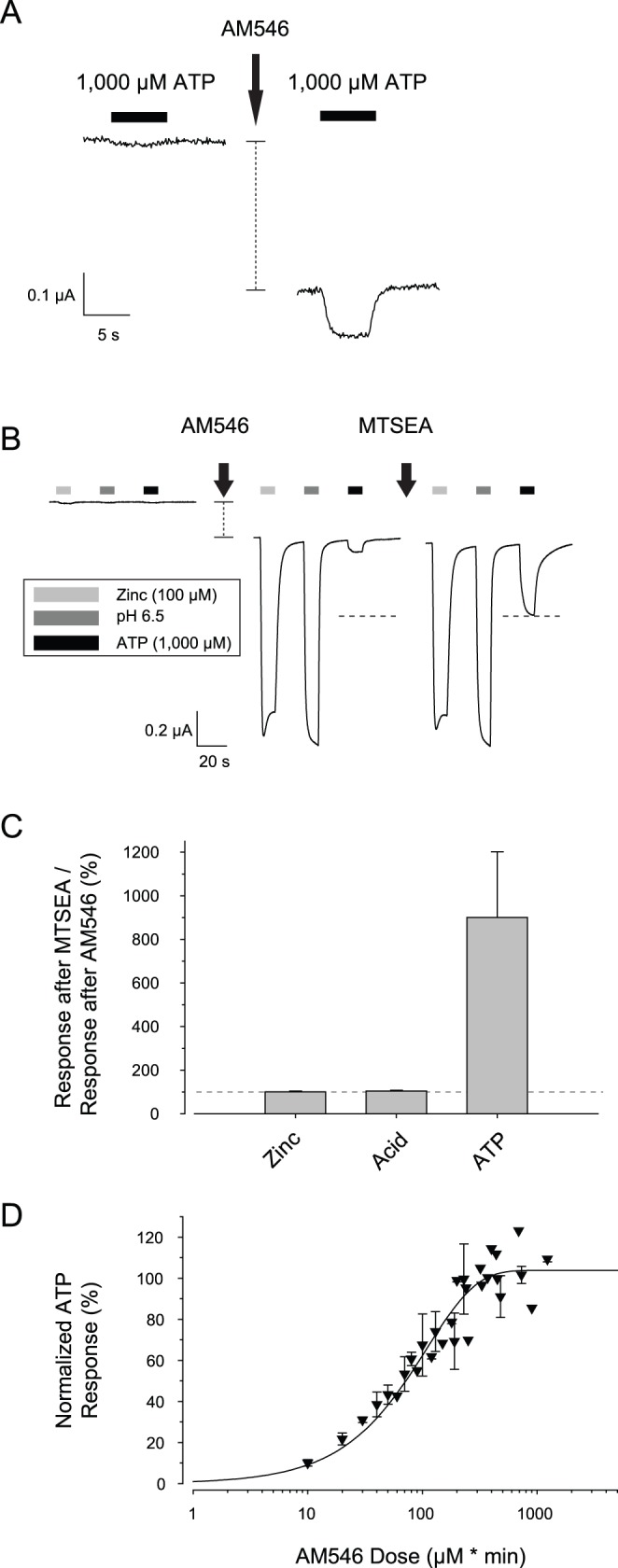
Zinc and acidic pH activate AM546-treated K69C receptors to a greater degree than ATP alone. A: Response of a K69C expressing oocyte to 1,000 µM ATP before and after AM546 (50 µM, 5 min). Traces were *not* baseline-corrected to emphasize the drop in holding current (vertical *dashed* line) after AM546 exposure. B: Effect of MTSEA challenge after treatment with AM546. The vertical dashed line indicates the drop in holding current after AM546. The horizontal dashed lines indicate the amplitude of the ATP response after MTSEA. C: Summary of a series of experiments as in B. The data are plotted as the responses after MTSEA normalized to the responses after AM546. The horizontal dashed line indicates 100% (i.e. no change in response after MTSEA). (N = 8). D: Effect of AM546 dosage (concentration * duration of exposure) on inward currents evoked by 1,000 µM ATP.

If AM546 had covalently bound to K69C in all three ATP binding sites and occluded them, responses to ATP would be predicted to be absent. This was not the case. Rather, the response to 1,000 µM ATP was usually much larger after AM546 treatment (Fig. 6A). The ATP concentration-response relation of AM546-modified K69C receptors was so far right-shifted that osmotic factors prevented us from raising the ATP concentration high enough to accurately assess it, but detectable responses were occasionally observed at 200 µM ATP (although the average response of −10±11 nA; N = 7 was not significantly different from zero) and ATP responses greater than −100 nA were usually present at 1,000 µM. The observation that AM546-treated K69C receptors were responsive to ATP suggested that at least one of the three ATP binding sites remained unmodified. To test this idea, we treated K69C oocytes with AM546, assayed the zinc, acidic pH and ATP responses, and then treated them with MTSEA, which would be expected to greatly increase the ATP affinity of any sites where 69C remained free after AM546 treatment (see Fig. 1A). MTSEA treatment of K69C receptors modified by AM546 led to a substantial increase in the response to 1,000 µM ATP, with no change in the response to zinc or acid jumps in the absence of ATP (Fig. 6 B–C). Moreover, the amplitude of the responses to 100 µM zinc were greatly potentiated by the presence of 1,000 µM ATP after MTSEA (without ATP 0.39±0.06 µA, with ATP 2.74±0.47 µA, N = 6).

One possible reason that the ATP responses were present might be because the AM546 incubation was not sufficiently long to saturate all the mutant cysteines. If this were the case, then at long enough time points, the ATP response would approach zero. To test this hypothesis, we incubated K69C oocytes in variable doses of AM546 (defined as µM*min, because both concentration and time were varied) and assessed the response to 1,000 µM ATP. We found that the response reached a maximum and did not fall back to zero (Fig. 6D). Furthermore, the dose required to get a maximal ATP response was very similar to the dose used in Fig. 3 (5 min of 100 µM AM546) that produced maximal changes in the zinc and pH responses. A plausible explanation for this result is that covalent binding of the first or second AM546 molecule causes the receptor to assume a conformation which prevents the binding of AM546 to the remaining free cysteines.

### Enhanced Responses to Zinc by a Double Mutant rP2X2 Receptor

**Figure 7:**
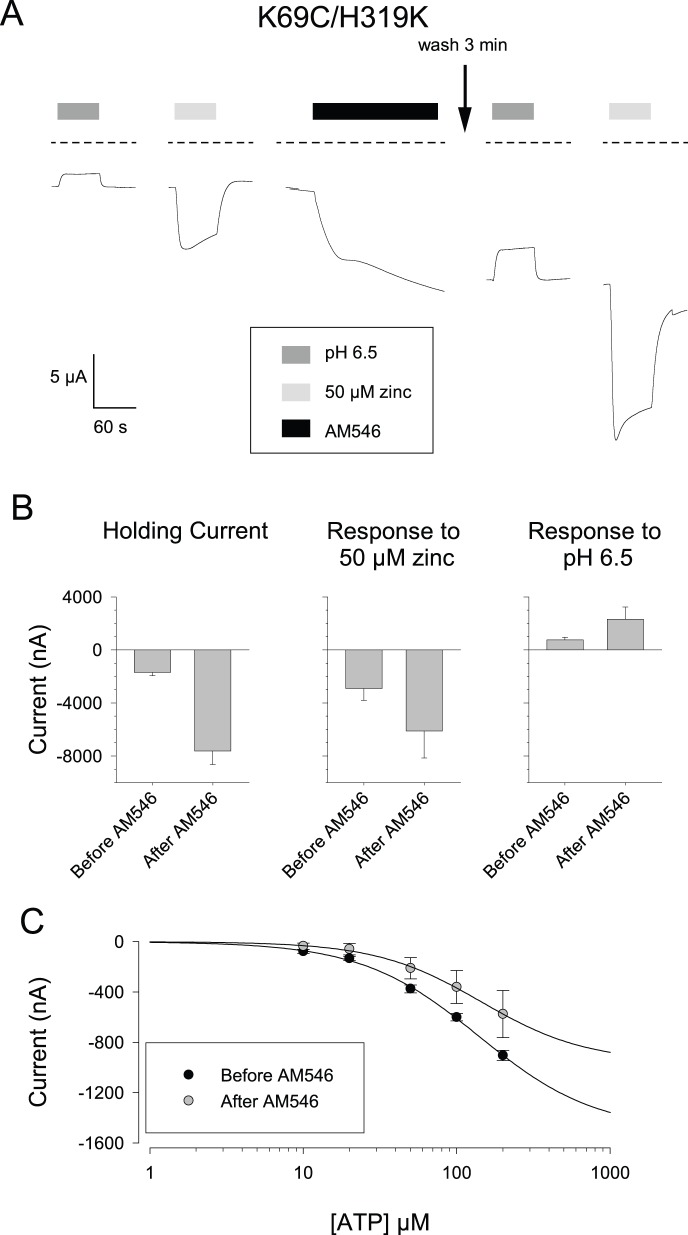
Effects of AM546 on the K69C/H319K double mutant. A: Responses to acidic pH and zinc before and after AM546 treatment. The dashed lines indicate the 0 current level, and indicate that there was a large inward holding current prior to any treatment. B: Average responses for a series of cells studied as in A. (N = 16 for holding current, 8 for zinc and 4 for pH 6.5). In contrast to the other figures, inward currents are plotted as downward deflections, to emphasize the different signs of the responses to zinc and acidic pH in this mutant. C: ATP concentration-response relations before and after AM546 treatment (N = 4).

In wild type rP2X2, a lysine substituted at position H319 strongly suppressed pH potentiation and enhanced responsiveness to ATP [17]. We therefore examined the responses of the K69C/H319K double mutant before and after AM546 treatment. Even before AM546 was added, many properties of this mutant were dramatically different from K69C (Fig. 7A). First, oocytes expressing this double mutant had much larger inward holding currents (−1.7±0.3 µA, N = 16), than oocytes expressing K69C (which typically were less than −0.3 µA), indicating that a substantial number of double mutant channels were open in the absence of exogenous ATP. Second, unlike K69C, either acidic pH alone or 50 µM zinc alone produced a response, however, they were in opposite directions (shift to pH 6.5 = +0.8±0.2 µA; N = 4; 50 µM zinc = −2.9±0.9 µA, N = 12). It seems likely that the “outward” current in response to acidic pH was actually a suppression of the standing inward current, as the total current at −50 mV remained inward at pH 6.5. When AM546 was added, there was a very large increase in the holding current (−5.9±0.8 µA, N = 16) so the total inward holding current was over −7.5 µA, This inward current was sustained over the duration of the observation period. From this new baseline, a shift to acidic pH gave an outward shift of over +2 µA, while 50 µM zinc produced an additional inward current of over −6 µA (Fig. 7B).

The K69C/H319K double mutant responded to ATP both before and after AM546 treatment (Fig. 7C). When ATP concentrations up to 200 µM were tested, the currents at each concentration were somewhat smaller after AM546 treatment, but the EC_50_ was similar before and after AM546 (approximately 145 µM, Fig. 7C). Comparison of figures B and C demonstrate that in this double mutant, the maximum inward current elicited by ATP was substantially smaller than the current zinc could elicit. The ATP concentration response curve for the double mutant also indicates that the large holding current was likely due to channels opening spontaneously, rather than by ATP leaking out of the oocyte, as there was very little ATP dependent current below 10 µM, while the maximal concentration endogenously released ATP can reach is about 2 µM (see Discussion).

### Specificity of the AM546 Effect

**Figure 8:**
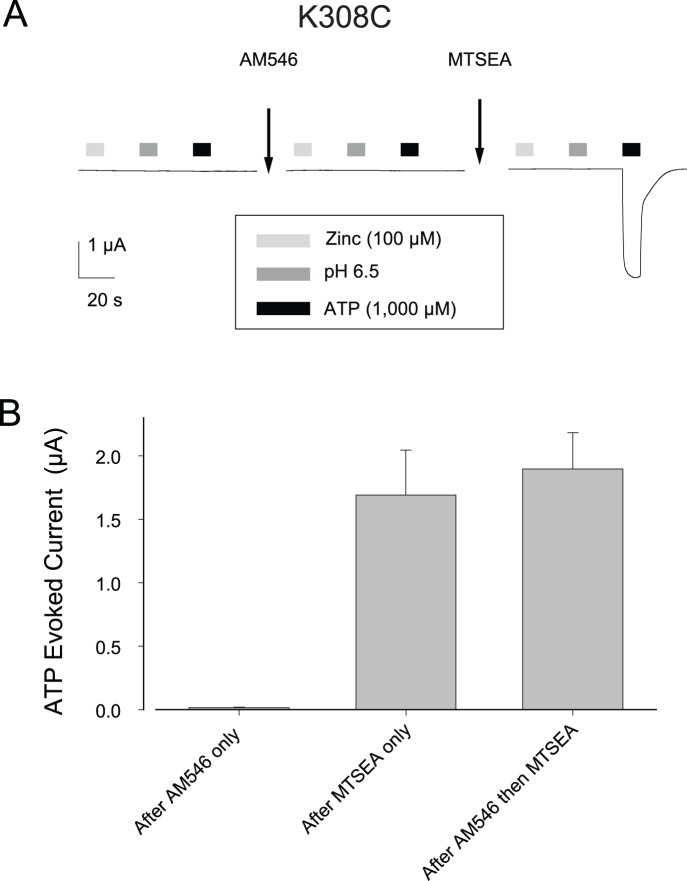
Lack of effect of AM546 on K308C. A: Responses of an oocyte before and after AM546 (50 µM, 15 minutes). The cell was then treated with MTSEA to test whether the cysteines had been modified by the AM546 treatment. B: Average ATP responses for a series of cells studied as in A or control cells that received MTSEA only. There was no response to zinc or pH 6.5 before or after AM546 or MTSEA treatment.

To explore the specificity of the AM546 effect, we tested whether this compound could activate receptors bearing the K308C mutation because K308 is also a critical element of the ATP binding pocket [25], [27], [29], [30], [33], [37]. Like K69C oocytes, oocytes expressing K308C were unresponsive to 100 µM zinc, pH 6.5 solution, and 1,000 µM ATP (Fig. 8A). The average currents (in nA) were: I_zinc_, −5±4; I_pH 6.5_, 3±1; I_ATP_, 5±1 (N = 6). However, in sharp contrast to the results at K69C, treatment with AM546 (50 µM, 15 min, Fig. 8A) resulted in no change in these responses (I_zinc_, −6±4; I_pH 6.5_, 2±3; I_ATP_, 14±4, N = 6). After exposure to AM546, MTSEA was able to modify K308C expressing oocytes to an extent similar to oocytes not pre-treated with AM546 (Fig. 8B), indicating that the reason that AM546 had no effect was that the maleimide could not access the free cysteine of K308C.

As a second way to assess the specificity of the AM546 responses, we applied three other large cysteine-reactive Alexa compounds (Structures are shown in Fig. S3) to the K69C receptors and tested for responses to zinc, pH 6.5 and ATP. In each case, the initial test was followed by a challenge with AM546 or MTSEA to determine whether K69C had been successfully modified. Alexa Fluor 488 C_5_-maleimide, Alexa Fluor 568 C_5_-maleimide and Alexa Fluor 594 C_5_-maleimide all could bind to, but not activate, K69C receptors (Table 1). These results demonstrate that activation of the K69C channel by AM546 is not simply due to the large size of the modifying group.

**Table 1:** Effect of other Alexa maleimides on K69C responses.

Test compound	Challenge compound	Response after challenge compound (nA)			
		100 µM Zn	pH 6.5	1,000 µM ATP	N
MTSEA	None-Control			−715±353	3
AM-546	None-Control	−114±37	−272±87	−144±48	8
AM-488	AM-546	−10±9	−16±5	−10±3	4
AM-568	MTSEA	−9±4	−25±0	−41±9	3
AM-594	MTSEA	−2±1	−7±5	−26±7	3

In these experiments K69C expressing oocytes were first exposed to the test compound (50 µM, 5 minutes), then tested for responses to zinc, pH 6.5 and ATP, then exposed to the challenge compound (50 µM, 5 minutes). The purpose of the challenge compound application was to verify that the test compound had successfully bound to the K69C receptors. If it had not, then responses after the challenge compound would have been similar to the challenge compound given alone. Data for the MTSEA control are also shown in Figure 1 and data for the AM546 control are also shown in Figure 2 and Figure 6B–C.

### Suramin Responses of K69C Expressing Oocytes after AM546 Treatment

**Figure 9:**
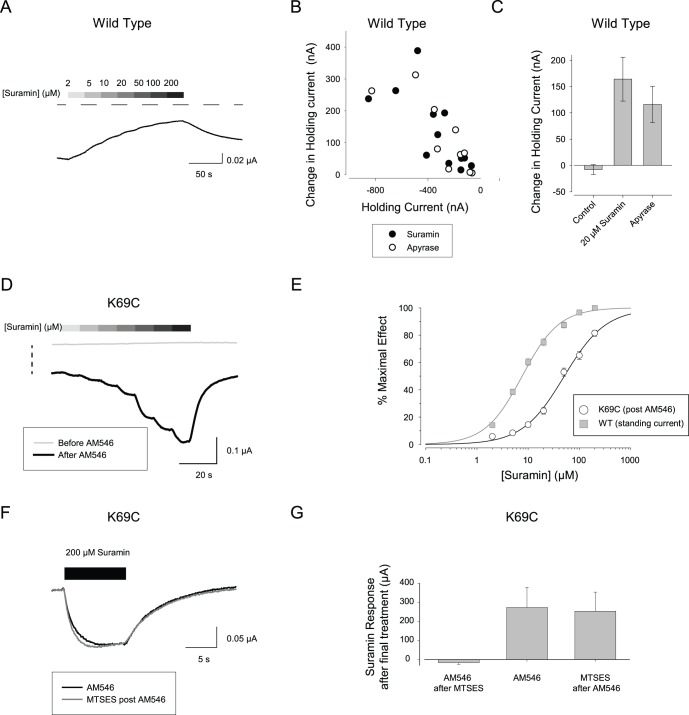
Responses of AM546-treated K69C receptors to suramin. A: Effect of suramin on an oocyte expressing wild type rP2X2 when applied without exogenous ATP. The horizontal dashed line indicates the 0 current level. B: Correlation between the initial holding current of oocytes expressing wild type rP2X2 and the amplitude of the outward current in response to suramin (20 µM) or apyrase (1 mg/ml) C: Average amplitude of the outward current in response to suramin, apyrase, or recording solution alone applied from a separate barrel of the solution switcher (control). D: Effect of suramin on an oocyte expressing K69C before AM546 (thin gray trace) and after AM546 (thick black trace). The two traces were *not* baseline corrected to indicate the drop in holding current caused by AM546 (vertical dashed line). E: Concentration-response plots of suramin on wild type rP2X2 and AM546-treated K69C receptors (N = 6). F: Response of a K69C oocyte to 200 µM suramin after treatment with AM546 and then retested after exposure to MTSES. G: Results of a series of experiments as in F. The “AM546 after MTSES” group represents a control to verify that the dose of MTSES given to the “MTSES after AM546” experimental group was sufficient to occupy all free cysteines.

For wild type rP2X2 receptors, suramin acts as a relatively potent antagonist. However, for some mutant receptors in which open probability has been enhanced, suramin can act as an agonist [15] and we wondered whether this might be true of AM546-modified K69C receptors. When suramin was applied to oocytes expressing wild type rP2X2 in the absence of exogenous ATP, small concentration-dependent outward currents were observed (Fig. 9A). The IC_50_ for this suramin response was 8.3±1.1 µM and the Hill coefficient was 1.2±0.1 (N = 11). Evidence that these outward currents were due to the inhibition of a standing current elicited by the basal release of small amounts of ATP from the oocyte acting on the rP2X2 receptors was that the suramin response was absent in uninjected oocytes (average response −1.3±1.9 nA, N = 14) and that the maximal outward current at saturating suramin concentration was highly correlated with the inward holding current prior to suramin application (i.e. oocytes with small holding current had little or no suramin activated outward current (Fig. 9B). A similar result was obtained when the standing current was inhibited with the ATP degrading enzyme apyrase (Fig. 9B, C). For cells with maximal currents to saturating ATP of about −20 µA, the outward suramin currents due to inhibition of basal ATP were in the range of 10–500 nA. Given this range of currents and using typical values for the EC_50_ (20 µM) and Hill Coefficient (1.5) for wild type rP2X2, the range of basal ATP levels is 0.1–2 µM.

Prior to AM546 treatment, K69C expressing oocytes showed no response to suramin applied without exogenous ATP (Fig. 9D, thin trace). After AM546 treatment, K69C expressing oocytes exhibited concentration-dependent inward currents in response to suramin (Fig. 9D, thick trace). The maximal response averaged −450±51 nA and the other parameters of the concentration-response relation for suramin activation (Fig. 9E) were EC_50_ 52±5 µM and Hill coefficient of 1.1±0.1.

The results shown in Fig. 6 demonstrated that AM546 does not modify all the mutant cysteines of K69C receptors, as a subsequent MTSEA challenge greatly potentiated the responses to ATP. We were interested in whether suramin agonist activity required that the ATP binding sites that were not occupied by AM546 have unmodified cysteines. To test this idea, we applied AM546, MTSES and MTSEA in several different orders (Fig. 9F,G) and measured the responses to 200 µM suramin and 1,000 µM ATP. As expected based on the observation that pre-application of MTSES occluded an MTSEA challenge (Fig. S1), when MTSES was applied before AM546 (Fig. 9G) there was no suramin agonist response, presumably because the MTSES occupied the free cysteines in all three ATP binding sites so AM546 could not bind to them. In contrast, when MTSES was applied after AM546, the inward currents activated by suramin were as large as the currents after treatment with AM546 only (Fig. 9F,G). MTSES applied after AM546 also caused no change in the response to ATP (94±4% of the response to ATP after AM546 only, N = 6), so one potential explanation for the lack of change in suramin and ATP responsiveness was that MTSES failed to bind to the cysteines that remained free after AM546 treatment. However this was not the case, because in oocytes from the same frog, MTSES treatment greatly occluded the ability of a subsequent MTSEA challenge to increase the currents in response to ATP (AM546 then MTSEA, 501±191%, N = 4; AM546 then MTSES then MTSEA, 135±10%, N = 6). We conclude that the agonist activity of suramin and ATP on the AM546 modified channels can still occur under conditions when most ATP binding sites are covalently modified at 69C. Consistent with this interpretation, it has been reported that the P2X1 mutant homologous to K69A has a normal IC50 for suramin [28].

## Discussion

Our principle finding is that AM546 was able to modify K69C receptors so that some opened in the absence of exogenous ATP, and many more opened in response to zinc, acidic pH or suramin, which normally only modulate channel activity in the presence of ATP. It should also be noted that the maximum currents produced by these modulators were less than 3 µA, which represents only about 5–10% of the current that could be produced when these receptors are modified by MTSEA and then activated by saturating ATP. Our results indicate that the inward shift in holding current represented opening of rP2X2 channels in the absence of ATP binding rather than responses to the low level of endogenous ATP released by oocytes, because the basal ATP concentration was shown to be 0.1–2 µM, but after AM546 modification of K69C no responses to exogenous ATP were observed at concentrations below 200 µM. In wild type rP2X2, zinc is unable to open the channels in the absence of ATP [21], [22], but in our experiments the basal ATP concentration was sufficient to allow small zinc dependent currents. However, the unmodified K69C receptors have very low ATP potency, and inward currents in response to zinc, acidic pH or suramin were never observed in this mutant.

Apparently the change in free energy provided by binding zinc, protons or suramin is too small to open the wild type channels unless additional free energy is provided by ATP, and AM546 has a similar but less efficacious action on K69C channels. Some other mutant channels also show enhanced responses to these modulators. T339S mutants show basal openings in the absence of ATP [15], and similar to the AM546 activated K69C channels, can be activated in an ATP independent manner by suramin [15] or zinc [22]. We also showed here that the K69C/H319K double mutant channels can open, and are zinc sensitive without the need to add ATP or AM546. In this case, much larger zinc responses were produced after AM546 treatment.

### Structural Features that Might Account for the Actions of AM546

**Figure 10:**
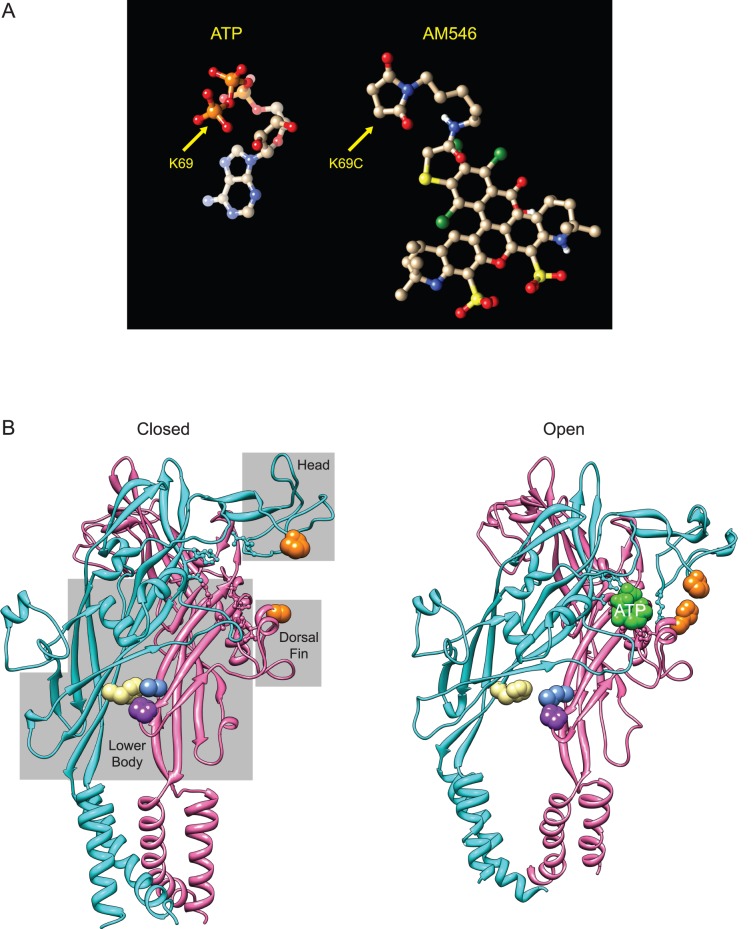
Structural features likely contributing to activation of AM546 modified K69C channels by zinc or pH. A: Structure of ATP folded as it sits in the binding site of zebrafish P2X4.1, and structure of AM546 with the linker folded back upon itself. B: Structure of P2X4.1 in the closed and open (ATP-bound) states. Only two of the three subunits are illustrated. Residues that bind ATP are shown in ball and stick format. The gray boxes superimposed on the closed state indicate domains of the receptor named according to the dolphin model of Kawate and Gouaux [32]. Left: The closed state structure of zP2X4.1 (PDB 4DW0) with positions homologous to key residues of rP2X2 colored. Orange indicates the location of the histidines of the potentiating zinc binding site of rP2X2, which are P125 (cyan subunit) and H219 (pink subunit) in zP2X4.1. Beige is the location of F327 (of the cyan subunit), which is homologous to rP2X2 H319. Blue (L64) and purple (P199) indicate the residues (of the pink subunit) that bind to F327 in the closed state. Right: The ATP bound open state structure of zP2X4.1 (PDB 4DW1). Residues are colored as in A. The ATP at the interface between the two illustrated subunits is also shown (green). Note that the side group of H219 has been rotated from its position in 4DW1 to emphasize the close apposition of the orange histidines that is possible in rP2X2 when zinc is present.

Recently, a crystal structure of zP2X4.1 in an ATP-bound, open state was reported [33] that complements the earlier ATP-free, closed channel structure [32] and comparison of the two structures strongly suggested how this channel opens in response to binding ATP [33]. The new structure confirms that the zP2X4.1 residues that are the homologs of rP2X2 K69 and K308 (K70 and K316) directly interact with ATP across the subunit interface, as do nine other residues (three on the same “A” subunit as K308, and six on the same “B” subunit as K69). Because most of these ATP binding residues are present in both rP2X2 and zP2X4.1, it seems likely that the conformational changes during opening of these two subunits are very similar, and so the zebrafish P2X4.1 open and closed structures suggest potential explanations for several of our observations. Gouaux and collaborators analogized the domains of P2X receptors to the body of a dolphin. In discussing these results, we will use the rP2X2 numbering for key residues. The interface between the bottom of the “upper body” of one subunit (N288, R290, K308) and the top of the “lower body” of the adjacent subunit (K69, K71,K193) serves as an ATP dependent pivot point. When the phosphates of ATP interact with these residues, it positions the purine ring so that the “head” of the “A” subunit and the “dorsal fin” of the “B” subunit can close around it. The “left flipper” also rotates to bring additional residues in contact with the ATP. Because the dorsal fin and left flipper are both tightly coupled to the lower body, when they move the lower body flexes outward, splaying open of the transmembrane domains that attach to it to produce an open channel. AM546 is a much larger molecule than ATP (Fig. 10A). The linker between the maleimide and the fluorochrome is extremely flexible, so it is unclear how AM546 folds into the ATP binding site to promote opening once a covalent bond forms between the maleimide and K69C.

Comparison of the closed state and open state structures suggests how zinc or acidic pH was able to produce channel opening of rP2X2 when AM546 was bound, and why the H319K mutation strongly promoted channel opening. We presume that binding AM546 to 69C slightly increases domain closure (see below), modestly increasing entry into the open state and so producing a small inward current. The zinc binding site of rP2X2 consists of H120 in the head and H213 in the dorsal fin. The residues at the homologous position of zP2X4.1 (P125 and H219) can come much closer together in the open state (Fig. 10A), exactly as expected if zinc stabilizes the open state of rP2X2 by promoting domain closure. Additional evidence in support of this mechanism has recently been reported [22]. The likely mechanism of action of a positive charge at H319 is quite different. In the zP2X4.1 closed state, the residue at this position (F327) makes cross subunit bonds near the bottom of the lower-body (to L64 and P199), which need to be broken in order for the channel to open. H319 of rP2X2 would be expected to make similar bonds to E63 and P194. It seems likely that adding a positive charge at this location (whether by protonation or mutation) destabilizes the closed state by disrupting these bonds. Interestingly, in human P2X2 the homologous residues (E75 and H330) plus some nearby histidines are required for high potency zinc inhibition [38] presumably because zinc binding near this site stabilizes the closed state.

We found that AM546 could bind to K69C, but not to K308C receptors, while MTSEA could bind to either position. The likely explanation for this is that the only part of K308 that is exposed in the binding site is its terminal amine, and when a shorter cysteine replaces K308, the maleimide ring cannot fit into the crevice between N288 and R290 to reach the exposed SH group, while the more linear MTSEA can. In contrast, almost the entire length of K69 is exposed within the binding site, with the α, ß and γ phosphates of ATP wrapping around this lysine in a conformation not seen in any other known ATP binding site. Three other Alexa maleimides could also bind to 69C, but were unable to promote channel opening. The ATP bound structure does not provide a simple explanation of why this was the case, but several differences seem potentially relevant (Fig. S3). Most obviously, the chemical structure of each fluorochrome is different (although the basic ring structures of AM568 and AM594 are very similar to AM546). As each fluorochrome is linked to K69C by a relatively long, highly flexible, linker, every position at which AM546 differs from the other Alexa dyes might potentially be a site that interacts with the residues in the head or dorsal fin to produce modest domain closure. Alternatively, a chemical feature of AM546 that is shared with some of the other Alexa dyes might be responsible for promoting domain closure, but differences between the linkers may explain why only AM546 interacts productively. AM546 is a single isomer with the linker attached at the 6 position, while AM488, AM568 and AM594 are mixed isomers, with the linker attaching to the fluorochrome at either the 5 or 6 position. Perhaps more significantly, AM488, AM568 and AM594 all have the same linker (maleimide-(CH_2_)_5_-NH-C = O), but the linker on AM546 is two atoms longer (maleimide-(CH_2_)_5_-NH-C = O-CH_2_-S), which might allow the AM546 fluorochrome to get to locations the others cannot reach.

## Supporting Information

Figure S1 **Specificity of MTSEA effects.** All MTS compounds were tested at 1 mM. The duration of the incubation in the test compound was 1 minute for K69C and 15 minutes for K308C. The duration of the subsequent MTSEA challenge was 1 minute. A–B: ATP responses from K69C and K308C expressing oocytes after exposure to MTSES and then tested again after a subsequent exposure to MTSEA. C–D: Bar graph summaries of a series of oocytes treated as in A and B (N = 4–5). The insets show the same data on an expanded scale. The asterisks in the inset of D indicate a significant increase in current after the MTSEA challenge.
(EPS)

Figure S2 **Responses to zinc and acidic pH on K69C receptors modified with AM546 are super-additive.** The dashed lines in each panel indicate the sum of the responses to zinc and acidic pH. A: Representative trace of a wild type rP2X2 expressing oocyte under high activation conditions. B: Representative trace of a wild type rP2X2 expressing oocyte under low activation conditions. C: Representative trace of a K69C expressing oocyte after AM546 treatment. There was no exogenous ATP. D: Summary of results from a series of experiments as in A–C. The summation index was defined as (response to zinc alone + response to pH 6.5 alone)/(response to simultaneous application of zinc and pH 6.5). A summation index of 1.0 (dashed line) would indicate linear summation (WT, N = 5; K69C, N = 3).
(EPS)

Figure S3 **Chemical structures of the Alexa maleimides tested on K69C.** All consist of a maleimide, linker and fluorochrome, but note that the linker of AM546 is different than the others, and that AM488, AM568 and AM594 are each a mixture of two isomers with the linker bound to either the 5 or 6 carbon, while AM546 is a single isomer.
(EPS)
